# Novel Association of ABO Histo-Blood Group Antigen with Soluble ICAM-1: Results of a Genome-Wide Association Study of 6,578 Women

**DOI:** 10.1371/journal.pgen.1000118

**Published:** 2008-07-04

**Authors:** Guillaume Paré, Daniel I. Chasman, Mark Kellogg, Robert Y. L. Zee, Nader Rifai, Sunita Badola, Joseph P. Miletich, Paul M. Ridker

**Affiliations:** 1 Center for Cardiovascular Disease Prevention, Brigham and Women's Hospital, Harvard Medical School, Boston, Massachusetts, United States of America; 2 Donald W. Reynolds Center for Cardiovascular Research, Brigham and Women's Hospital, Harvard Medical School, Boston, Massachusetts, United States of America; 3 Department of Laboratory Medicine, Children's Hospital, Harvard Medical School, Boston, Massachusetts, United States of America; 4 Amgen, Inc., Cambridge, Massachusetts, United States of America; The University of Queensland, Australia

## Abstract

While circulating levels of soluble Intercellular Adhesion Molecule 1 (sICAM-1) have been associated with diverse conditions including myocardial infarction, stroke, malaria, and diabetes, comprehensive analysis of the common genetic determinants of sICAM-1 is not available. In a genome-wide association study conducted among 6,578 participants in the Women's Genome Health Study, we find that three SNPs at the ICAM1 (19p13.2) locus (rs1799969, rs5498 and rs281437) are non-redundantly associated with plasma sICAM-1 concentrations at a genome-wide significance level (P<5×10^−8^), thus extending prior results from linkage and candidate gene studies. We also find that a single SNP (rs507666, P = 5.1×10^−29^) at the ABO (9q34.2) locus is highly correlated with sICAM-1 concentrations. The novel association at the ABO locus provides evidence for a previously unknown regulatory role of histo-blood group antigens in inflammatory adhesion processes.

## Author Summary

Soluble Intercellular Adhesion Molecule 1 (sICAM-1) is an inflammatory marker that has been associated with several common diseases such as diabetes, heart disease, stroke, and malaria. While it is known that blood concentrations of sICAM-1 are at least partially genetically determined, our current knowledge of which genes mediate this effect is limited. Taking advantage of new technologies allowing us to interrogate genetic variation on a whole genome basis, we found that a variation in the ABO gene is an important determinant of sICAM-1 blood concentrations. Since the ABO gene is responsible for the ABO blood groups, this discovery sheds light on a new role for blood groups and offers novel mechanisms to explain the association between sICAM-1 blood concentrations and various common diseases.

## Introduction

ICAM-1 is a member of the immunoglobulin superfamily of adhesion receptors and consists of 5 immunoglobulin-like extracellular domains, a transmembrane domain and a short cytoplasmic domain. ICAM-1, present on endothelial cells, serves as a receptor for the leukocyte integrins LFA-1 (lymphocyte function-associated antigen-1) and Mac-1 (CD11b/CD18), facilitating leukocyte adhesion and migration across the endothelium [1]. A soluble form of ICAM-1 (sICAM-1) is found in plasma and consists of the extra-cellular domains of ICAM-1. Although the process leading to the formation of sICAM-1 is not entirely clear, sICAM-1 is thought to be shed from the cell membrane via proteolytic cleavage of ICAM-1. Because sICAM-1 binds to LFA-1, it is capable of inhibiting lymphocyte attachment to endothelial cells [2]. Furthermore, sICAM-1 has been shown to bind human rhinoviruses, the etiologic agent of 40–50% of common colds, and to inhibit rhinovirus infection *in vitro* [3]. Likewise, a circulating fragment of sICAM-1 binds to erythrocytes infected with *Plasmodium falciparum*, the etiologic agent of malaria [4] (MIM 611162). Finally, plasma concentration of sICAM-1 has been shown to provide unique predictive value for the risk of myocardial infarction (MIM 608446), ischemic stroke (MIM 601367), peripheral arterial disease (MIM 606787) and noninsulin-dependent diabetes mellitus (MIM 125853) in epidemiological studies [5]–[7].

Despite relatively high heritability estimates (from 0.34 to 0.59) [8],[9] for sICAM-1, few genetic variants are known to influence its concentrations. Two recent linkage studies have shown evidence for genetic association at the ICAM1 (GeneID 3383) locus (19p13.3-p13.2) [8],[9] and two candidate SNPs within the extracellular domains of ICAM-1 itself, G241R (rs1799969) and K469E (rs5498), have been correlated with circulating sICAM-1 levels [10],[11]. By contrast, a recent genome wide association study (GWAS) from the Framingham investigators involving 1006 participants and 70,987 SNPs revealed no association reaching a genome-wide level of significance, including the ICAM1 locus itself, although this study had no genetic marker within 60 kb of the gene [12]. To more comprehensively explore this issue, we performed a larger GWAS, evaluating 336,108 SNPs in 6,578 apparently healthy women.

## Methods

### Study Sample and sICAM-1 Measurements

All participants in this study were part of the Women's Genome Health Study (WGHS) [13]. Briefly, participants in the WGHS include American women from the Women's Health Study (WHS) with no prior history of cardiovascular disease, diabetes, cancer, or other major chronic illness who also provided a baseline blood sample at the time of study enrollment. The WHS is a recently completed 2×2 randomized clinical trial of low-dose aspirin and vitamin E in the primary prevention of cardiovascular disease and cancer. For all WGHS participants, EDTA anticoagulated plasma samples were collected at baseline and stored in vapor phase liquid nitrogen (−170°C). Circulating plasma sICAM-1 concentrations were determined using a commercial ELISA assay (R&D Systems, Minneapolis, Minn.); the assay used is known not to recognize the K56M (rs5491) variant of ICAM-1 [14] and the 22 carriers of this mutation were therefore excluded from further analysis. This study has been approved by the institutional review board of the Brigham and Women's Hospital. Additional clinical characteristics of these subsets are provided in Table S1.

### Genotyping

Genotyping was performed in two stages, a first sample being used to discover new associated loci and the second sample being used to validate them by replication. These two samples were genotyped independently of one another in two batches. The first (WGHS-1) and second (WGHS-2) batches included 4,925 and 2,056 self-reported Caucasian WGHS participants, respectively. No related individuals were detected when tested with an identity by state analysis [15].

Samples were genotyped with the Infinium II technology from Illumina. Either the HumanHap300 Duo-Plus chip or the combination of the HumanHap300 Duo and I-Select chips was used. In either case, the custom content was identical and consisted of candidate SNPs chosen without regard to allele frequency to increase coverage of genetic variation with impact on biological function including metabolism, inflammation or cardiovascular diseases. Genotyping at 318,237 HumanHap300 Duo SNPs and 45,571 custom content SNPs was attempted, for a total of 363,808 SNPs. Genetic context for all annotations are derived from human genome build 36.1 and dbSNP build 126.

SNPs with call rates <90% were excluded from further analysis. Likewise, all samples with percentage of missing genotypes higher than 2% were removed. Among retained samples, SNPs were further evaluated for deviation from Hardy-Weinberg equilibrium using an exact method [16] and were excluded when the P-value was lower than 10^−6^. Samples were further validated by comparison of genotypes at 44 SNPs that had been previously ascertained using alternative technologies. SNPs with minor allele frequency >1% in Caucasians were used for analysis. After quality control, 307,748 HumanHap300 Duo SNPs and 28,360 custom content SNPs were left, for a total of 336,108 SNPs. From the initial 4925 WGHS-1 and 2056 WGHS-2 individuals genotyped, 4582 WGHS-1 individuals and 2014 WGHS-2 individuals were kept for further analysis.

### Population Stratification

Because population stratification can result in inflated type I error, a principal component analysis using 1443 ancestry informative SNPs was performed using PLINK [17] in order to confirm self-reported ancestry. Briefly, these SNPs were chosen based on Fst >0.4 in HapMap populations (YRB, CEU, CHB+JPT) and inter-SNP distance at least 500 kb in order to minimize linkage disequilibrium. Different ethnic groups were clearly distinguished with the two first components. Out of 4582 WGHS-1 and 2014 WGHS-2 self-identified Caucasians, 12 and 6 were removed from analysis because they did not cluster with other Caucasians, leaving 4570 (WGHS-1) and 2008 (WGHS-2) participants for analysis, respectively. Two more analyses were undertaken to rule out the possibility that residual stratification within Caucasians was responsible for the associations observed. First, association analysis was done with correction by genomic control. This method estimates the average effect of population substructure in the sample (based on median T values) and accordingly corrects the test statistics [18]. Second, a principal component analysis [19] was performed in Caucasians (only) using 124,931 SNPs chosen to have pair-wise linkage disequilibrium lower than r^2^ = 0.4. The first three components were then used as covariates in the association analysis. As adjustment by these covariates did not change the conclusions, we present analysis among the WGHS-1 and WGHS-2 Caucasian participants without further correction for sub-Caucasian ancestry unless stated otherwise.

### Association Analysis

To identify common genetic variants influencing sICAM-1 levels, we first attempted to discover which loci significantly contributed to sICAM-1 concentrations in WGHS-1. Plasma concentrations of sICAM-1 were adjusted for age, smoking, menopause and body mass index using a linear regression model in R to reduce the impact of clinical covariates on sICAM-1 variance. The adjusted sICAM-1 values were then tested for association with SNP genotypes by linear regression in PLINK [17], assuming an additive contribution of each minor allele. A conservative P-value cut-off of 5×10^−8^ was used to correct for the roughly 1,000,000 independent statistical tests thought to correspond to all the common genetic variation of the human genome [20]. Replication of genome-wide significant associations was performed on adjusted sICAM-1 values from the replication sample (WGHS-2), using a Bonferroni correction to account for multiple hypothesis testing.

### Model Selection Algorithm

To further define the extent of genetic associations, a forward selection linear multiple regression model was used at the previously identified loci. Briefly, all genotyped SNPs within 100 kb of the most significantly associated SNP at each replicated locus and passing quality control requirements were tested for possible incorporation into a multiple regression model. In stepwise fashion, a SNP was added to the model if its multiple regression P-value was less than 10^−4^ (to account for all the SNPs being considered) and if it had the smallest P-value among all the SNPs not yet included in the model. This analysis was done on WGHS-1 individuals using adjusted sICAM-1 values.

We then proceeded to validate our multiple regression model in WGHS-2 samples. Using only the SNPs previously selected in WGHS-1, we added them in a multiple regression model in the same order as they were chosen in WGHS-1. We considered the model validated if each time a SNP was included in the model, its regression P-value was lower than 0.01 (to account for multiple testing) and the direction of effect consistent.

### Analytical Interference Assay

Plasma from A blood group individuals was mixed 1∶1 or 1∶2 with a monoclonal anti-A antibody (Ortho-Clinical Diagnostics, Rochester NY), and allowed to incubate 10 minutes or 60 minutes at room temperature, or 60 minutes or 12 hours at 4°C before assaying sICAM-1 levels by the standard technique. To exclude the possibility that the antibody itself interfered with the assay, the same procedure was repeated with plasma from O blood group individuals. Finally, plasma from O group individuals, which is expected to contain both anti-A and anti-B polyclonal antibodies, was mixed with plasma from A group individuals in 1∶1 ratio, again with incubation as above and measurement of sICAM-1 levels.

## Results/Discussion

**Figure 1:**
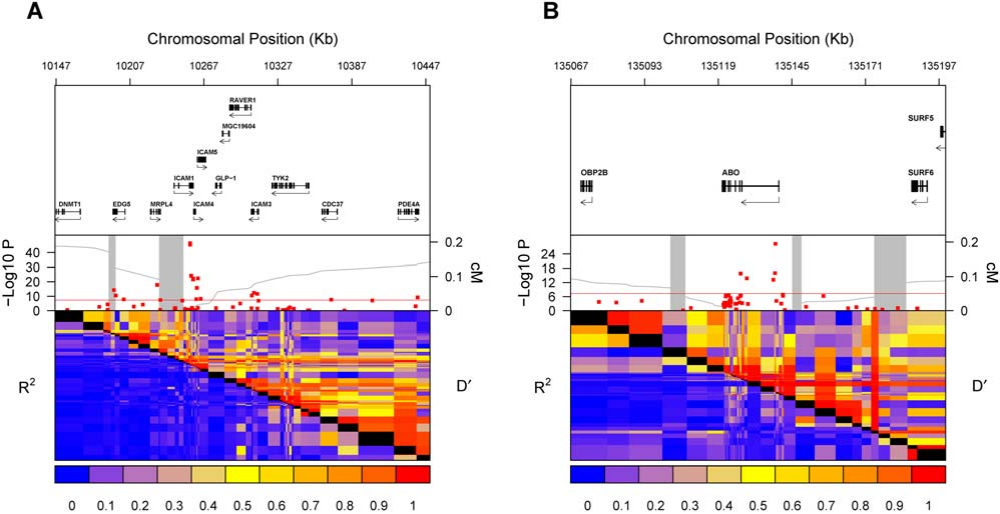
Genetic Context of Genome-Wide Significant Associations. Genomic context for each of two loci with genome-wide association with sICAM-1 levels. The ICAM1 locus (19p13.2) is shown in Figure 1-A and the ABO locus (9q34.2) in Figure 1-B. Upper panel: Genes from RefSeq release 25. Only one isoform is shown when multiple splicing variants are known. Middle Panel: SNPs are shown according to their physical location and P-values (red dots). Also shown is the genetic distance in cM from the lowest P-value SNP (light grey line) along with the position of recombination hotspots (light grey vertical bars). Recombination rates and hotspots are based on HapMap data, as described by McVean et al.[53] and Winckler et al. [54]. Lower panel: Pair wise linkage disequilibrium (D′ and R^2^) between SNPs based on WGHS data.

As shown in Table 1, 19 SNPs passed our stringent genome-wide significance threshold when tested in WGHS-1 individuals, clustering within two loci in the vicinity of the ICAM1 (19p13.2) and ABO (GeneID 28) (9q34.2) genes (Figure 1). The replication threshold in WGHS-2 was conservatively set at a 2-sided P-value of 0.002, applying a Bonferroni correction to account for 19 tests. Using this cutoff, we were able to replicate 17 of the 19 associated SNPs, including SNPs at both the ICAM1 and ABO loci. Only rs2116941 (19p13.2) and rs7256672 (19p13.2) did not replicate using this standard. Nevertheless, each of these SNPs had a P-value lower than 10^−9^ when tested on the combined sample (i.e. WGHS-1 and WGHS-2 pooled together). Among the replicated SNPs, only rs7258015 (19p13.2) deviated from Hardy-Weinberg equilibrium (p = 0.00007), but visual inspection of the raw genotyping signal for this SNP did not reveal any obvious artifact. Major and minor alleles are shown in Table S2.

**Table 1:** Genome-Wide Significant SNPs for sICAM1.

SNP	Locus	Position(kb)	Nearest Gene	Function	MAF^a^	HW^b^	WGHS-1		WGHS-2		Combined		Median sICAM-1 umol/L (n)		
							Beta	P-Value	Beta	P-Value	Beta	P-Value	A1/A1^c^	A1/A2^c^	A2/A2^c^
rs687621	9q34.2	135126.9	ABO	intron	0.34	0.08	−10.88	1.3E-11	−11.21	1.4E-06	−10.95	1.3E-16	343 (2856)	334 (2894)	328 (806)
rs687289	9q34.2	135126.9	ABO	intron	0.34	0.07	−11.00	8.2E-12	−10.81	3.5E-06	−10.91	1.7E-16	343 (2859)	334 (2887)	328 (804)
rs657152	9q34.2	135129.1	ABO	intron	0.37	0.13	−10.17	1.7E-10	−9.93	1.5E-05	−10.06	1.5E-14	343 (2652)	334 (2992)	331 (905)
rs500498	9q34.2	135138.5	ABO	intron	0.45	0.10	9.53	5.0E-10	9.53	2.4E-05	9.52	6.1E-14	332 (2017)	337 (3160)	347 (1358)
rs505922	9q34.2	135139.0	ABO	intron	0.34	0.05	−11.15	4.5E-12	−10.83	3.2E-06	−11.02	9.2E-17	343 (2876)	334 (2871)	328 (798)
rs507666	9q34.2	135139.2	ABO	intron	0.20	0.42	−18.12	1.1E-20	−16.93	5.9E-10	−17.73	5.1E-29	343 (4221)	328 (2055)	314 (268)
rs10409243	19p13.2	10194.0	EDG5	3′ UTR^d^	0.41	0.96	−10.93	4.4E-12	−8.22	2.6E-04	−10.11	5.4E-15	344 (2313)	335 (3158)	329 (1083)
rs2116941	19p13.2	10195.4	EDG5	3′ UTR^d^	0.20	0.76	−12.05	7.9E-10	−7.47	7.5E-03	−10.67	3.1E-11	341 (4209)	333 (2076)	320 (249)
rs8111930	19p13.2	10229.0	MRPL4	intron	0.12	0.27	18.88	4.4E-15	13.35	4.3E-05	17.10	1.3E-18	335 (5031)	346 (1369)	377 (102)
rs1799969	19p13.2	10255.8	ICAM1	NS^e^	0.12	0.34	−26.11	2.1E-28	−33.18	1.3E-21	−28.19	3.6E-47	343 (5117)	319 (1336)	304 (99)
rs5498	19p13.2	10256.7	ICAM1	NS^e^	0.43	0.15	13.88	6.2E-19	11.78	1.2E-07	13.22	4.8E-25	329 (2117)	338 (3123)	355 (1235)
rs923366	19p13.2	10258.2	ICAM1	3′ UTR^d^	0.44	0.71	13.07	5.7E-17	11.05	7.9E-07	12.46	2.5E-22	329 (2088)	338 (3202)	353 (1243)
rs3093030	19p13.2	10258.4	ICAM1	-	0.43	0.59	13.14	3.7E-17	11.41	2.7E-07	12.61	5.9E-23	329 (2109)	338 (3204)	353 (1243)
rs281440	19p13.2	10261.3	ICAM5	-	0.22	0.69	−13.39	7.2E-13	−10.99	4.9E-05	−12.69	1.5E-16	343 (4021)	331 (2223)	322 (314)
rs2075741	19p13.2	10262.1	ICAM5	intron	0.43	0.69	13.27	1.9E-17	11.40	3.2E-07	12.69	3.6E-23	329 (2104)	338 (3208)	353 (1240)
rs2278442	19p13.2	10305.8	ICAM3	intron	0.35	0.65	−9.44	1.0E-08	−7.66	7.7E-04	−8.88	3.1E-11	344 (2727)	334 (3007)	332 (815)
rs2304237	19p13.2	10307.6	ICAM3	NS^e^	0.22	0.49	10.83	1.2E-08	12.23	5.2E-06	11.26	3.9E-13	334 (3919)	342 (2129)	356 (313)
rs7258015	19p13.2	10310.4	ICAM3	NS^e^	0.22	0.00007	10.60	3.2E-08	12.73	8.6E-06	11.19	2.1E-12	334 (3873)	342 (2275)	356 (255)
rs7256672	19p13.2	10440.5	PDE4A	3′ UTR^d^	0.36	0.67	−9.06	2.7E-08	−6.36	5.8E-03	−8.24	6.3E-10	343 (2655)	337 (3049)	324 (845)

a MAF: Minor allele frequency based on the combined samples.

b HW: Deviation from Hardy-Weinberg equilibrium P-value based on the combined samples.

c A1: Major Allele; A2: Minor Allele; Median sICAM-1 values were determined on the combined samples.

d 3′ UTR: 3′ Untranslated Region.

e NS: Non-Synonymous Coding SNP.

We then applied our model selection algorithm in WGHS-1 individuals (see Methods) using 54 SNPs at 19p13.2 (ICAM1 locus) and 68 SNPs at 9q34.2 (ABO locus). As can be seen in Table 2, 3 out of 54 SNPs at 19p13.2 were selected by our algorithm and 1 out 68 SNPs at 9q34.2 was selected. All four SNPs selected in WGHS-1 were validated in WGHS-2. Pairwise linkage disequilibrium between these SNPs was low. For instance, r^2^ was lower than 0.35 between ICAM1 SNPs while it was lower than 0.002 between the ABO SNP rs507666 and the ICAM1 SNPs. Among these SNPs, there was no strong evidence for non-additive effects of the minor allele as judged by lack of significance for a likelihood ratio test comparing the additive regression model to an alternative genotype model with an additional degree of freedom. Interestingly, one of the four selected SNPs (rs281437) was non-significant in univariate analysis, illustrating that its inclusion in the model and significant association are conditional on the genotypes at rs5498 and rs281437. No gene-gene interaction was observed between ICAM1 and ABO SNPs. The 3 SNPs at 19q13.2 (ICAM1) collectively explained 6.9% of the total variance in sICAM-1 concentrations (pooling WGHS-1 and WGHS-2 together), whereas the ABO SNP rs507666 explained 1.5%. In comparison, clinical covariates accounted for 18.8% of the variance (Table 3), and together the candidate loci and the clinical variables accounted for 27.3% of total variance. It should be noted that the estimated effect sizes of the ICAM1 and ABO loci are minimums since the genotyped variants might not be the actual functional variants.

**Table 2:** Multiple Linear Regression Statistics of SNPs Retained by the Forward Model Selection Algorithm.

Locus	SNP	Nearest Gene	Function	MAF^a^	HW^b^	WGHS-1^c^		WGHS-2^c^		Combined^c^	
						Beta	P-Value	Beta	P-Value	Beta	P-Value
19p13.2	rs1799969	ICAM1	NS^d^	0.12	0.34	−41.2	<2.0E-16	−49.3	<2.0E-16	−43.5	<2.0E-16
	rs5498	ICAM1	NS^d^	0.43	0.15	30.4	<2.0E-16	28.3	<2.0E-16	29.7	<2.0E-16
	rs281437	ICAM1	3′ UTR^e^	0.30	0.47	11.2	1.3E-08	7.9	5.1E-03	10.1	3.2E-10
9q34.2	rs507666	ABO	intron	0.20	0.42	−16.8	<2.0E-16	−16.8	1.9E-10	−16.7	<2.0E-16

a MAF: Minor allele frequency based on the combined samples.

b HW: Deviation from Hardy-Weinberg equilibrium P-value based on the combined samples.

c All analyses were performed using adjusted sICAM1 values (see text for details). Beta coefficients and P-values were derived form a multiple linear model that included all 4 SNPs.

d NS: Non-Synonymous Coding SNP.

e 3′ UTR: 3′ Untranslated Region.

**Table 3:** Partition of sICAM-1 Variance According to Genetic and Clinical Variables.

Category	Variable	Variable R^2^	Category R^2^
Clinical Covariates	Age	0.012	0.188
	Body Mass Index	0.035	
	Menopause Status	0.006	
	Smoking	0.135	
9q34.2 (ABO) Locus	rs507666	0.015	0.015
19p13.2 (ICAM1) Locus	rs1799969	0.026	0.069
	rs5498	0.039	
	rs281437	0.005	
TOTAL			0.273

The 3 SNPs at the 19p13.2 (ICAM1) locus selected by our algorithm were also used in haplotype analysis using WHAP [21], as implemented in PLINK [17] (Table 4). The estimate of the proportion of variance attributable to haplotypes, as well as their regression coefficients, is consistent with the linear model of these same SNPs, reinforcing the adequacy of a strictly additive model to explain the association.

**Table 4:** Haplotype Analysis of rs1799969, rs5498 and rs281437 (19p13.2; ICAM1 Locus).

Haplotype			Frequency	Beta	P-Value
rs1799969	rs5498	rs281437			
A	G	G	0.12	−43.91	1.7E-95
G	A	G	0.27	−30.16	3.0E-78
G	A	A	0.30	−19.98	5.3E-37
G	G	G	0.32	Reference	-

Omnibus (3 df) p-value = 1.0E-124. WGHS-1 and WGHS-2 were combined for this analysis.

The ABO histo-blood group antigen is the most important blood group system in transfusion medicine. Using data from Seattle SNPs (http://pga.mbt.washington.edu) as well as from the Blood Group Antigen Mutation Database (www.ncbi.nlm.nih.gov), it can be demonstrated that rs507666 is a perfect surrogate for type A1 histo-blood group antigen. Moreover, using rs687289 as a marker for the O allele, rs8176746 for the B allele and rs8176704 for the A2 allele, complete blood group antigen phenotype can be re-constructed by haplotype analysis (no serotype data is available in WGHS). Imputed haplotypes perfectly fitted the pattern expected from the literature and their association with sICAM-1 is shown in Tables 5 and 6. The A1 allele is associated with the lowest sICAM-1 concentrations while the A2 allele is associated with low concentrations, intermediate between the A1 and O allele. In comparison, the B allele is associated with slightly higher concentrations than the O allele.

**Table 5:** Association of sICAM-1 Concentrations (µmol/L) with Histo-Blood Group Antigen Alleles (9q34.2; ABO Locus).

Allele	Genotype				Frequency	Beta
	rs8176746	rs8176704	rs687289	rs507666		
A1	C	G	A	A	0.20	−14.3
A2	C	A	A	G	0.07	−2.2
B	A	G	A	G	0.08	4.7
O	C	G	G	G	0.66	3.9

Omnibus (3 df) p-value = 2.0E-28. WGHS-1 and WGHS-2 were combined for this analysis.

**Table 6:** Mean (SD) Level of sICAM-1 (µmol/L) According to Predicted ABO Alleles.

		First Allele			
		A1	A2	O	B
Second Allele	A1	326 (71) N = 268	332 (71) N = 185	338 (77) N = 1656	352 (89) N = 182
	A2	-	346 (55) N = 33	352 (80) N = 537	366 (91) N = 56
	O	-	-	358 (81) N = 2859	356 (80) N = 592
	B	-	-	-	342 (79) N = 51

WGHS-1 and WGHS-2 were combined for this analysis. Mean (SD) values were derived from unadjusted sICAM-1 concentrations.

Because ABO histo-blood group antigens are known to vary in frequency among Caucasian sub-populations, we sought to investigate the potential effect of population stratification on the observed association even though adjustment of sICAM-1 values for the top ten components of our principal component analysis did not change our conclusions (see Methods). Visual inspection of the clustering pattern from the top two components confirmed a match with previously published work of sub-Caucasian stratification [22] (data not shown). Since these two components were reproducibly shown to correspond to a Northwest-Southeast European gradient [22] and the A1 allele follows such a gradient [23], we hypothesized that they would be tightly linked to A1 allele frequencies. Indeed, the second component showed evidence of association with A1 allelic frequencies (p = 2.5×10^−6^), while the first component was only weakly associated (p = 0.08). Nevertheless, neither the first nor second component was very tightly linked to sICAM-1 values (p = 0.69 and 0.0006 respectively with corresponding R^2^ of 3.8×10^−5^ and 0.0019), implying that stratification has no major effect on the sICAM-1 association. Furthermore, the weak association with the second component could be partially explained by the correlation with A1 alleles, with corrected P-value of 0.004 and R^2^ of 0.0013. Adjustment of sICAM-1 values for the first and second components did not substantially change the association between the A1 allele and sICAM-1 (unadjusted p = 5.1×10^−29^ and adjusted p = 5.5×10^−28^), demonstrating that stratification on a Northwest-Southeast European axis is not responsible for the association. We conclude that the data does not support the hypothesis that Northwest-Southeast sub-Caucasian stratification is responsible for the association of ABO variants with sICAM-1 concentrations since the A1 allele varies in frequency according to a Northwest-Southeast European axis while the slight variation in sICAM-1 among this same axis is at least partially dependent on the A1 allele. Indeed, there is no evidence in the literature that mean sICAM-1 concentrations vary at all among Caucasian sub-populations, and this lack of evidence is supported by an overall R^2^ of 0.005 (P-value of 0.0007) for the association between sICAM-1 concentrations and the top 10 principal components.

The Secretor phenotype (as defined by rs601338 on chromosome 19q13.33) and the Lewis antigen phenotype (as defined by rs812936 on chromosome 19p13.3) are additional important members of the histo-blood group antigen system. These were therefore tested for association with sICAM-1 levels as well as for interaction with rs507666. No significant effect was observed.

Although the sICAM-1 molecule itself is not known to bear the ABO histo-blood group antigen, this possibility could not be ruled out, especially given its extensive glycosylation [24],[25]. We therefore sought to exclude the remote chance that the association between A histo-blood group antigen and lower sICAM-1 values was the consequence of a lower affinity of the antibodies used in the sICAM-1 assay for sICAM-1 carrying the A antigen. In other words, if sICAM-1 does carry ABO histo-blood group antigen, then the allelic composition at the ABO locus could dictate the glycosylation status of the sICAM-1 molecule and possibly interfere with the immunoassay used. While there is no evidence that the two plasma proteins known to contain ABO histo-blood group antigen (von Willebrand factor and alpha 2-macroglobulin) [26] suffer from such analytical interference, immunoassays are potentially susceptible to differential glycosylation of their target protein [27]. We thus hypothesized that blocking the A antigen sites with either polyclonal or monoclonal antibodies would result in spuriously low sICAM-1 values if sICAM-1 does indeed carry ABO histo-blood group antigen and if the A antigen is located in the vicinity of one of the two antibody binding sites used by the immunoassay. No differential effects of the mixing procedures (see Methods) were observed suggesting that the A blood group antigen was not interfering with measurement of sICAM-1 levels. We therefore conclude that the genetic association of the ABO variant is not due to analytic interference. However, we can not exclude that sICAM-1 bears the ABO histo-blood group antigen.

Finally, we sought to assess the presence of other associations that did not pass our stringent genome-wide P-value cut-off. We therefore repeated the whole-genome association analysis on the combined sample (i.e. WGHS-1 and WGHS-2 pooled together). While no new locus was associated at a genome-wide level, rs9889486 had the lowest p-value (outside of 9q34.2 and19p13.2; p = 3.2×10^−6^) with a false discovery rate [28] of 0.03. This SNP is intronic to CCDC46 (GeneID 201134) (17q24.1), a gene whose function is not well characterized. Among other low p-value SNPs, we note rs1049728 (p = 1.3×10^−5^) with a false discovery rate of 0.08 and the 51^st^ most strongly associated SNP overall. This SNP is located in the 3′ untranslated region of RELA (GeneID 5970) (11q13.1), which is part of the NFKB signaling complex, arguably the most important known regulator of ICAM1 expression [29].

The non-synonymous coding ICAM1 SNPs rs1799969 (G241R) and rs5498 (K469E) were previously described as being associated with sICAM-1 levels[10],[11] whereas the association involving rs281437 is unreported. The later SNP is in the 3′ untranslated region of ICAM-1. Of interest, the minor allele of rs1799969 (arginine) is correlated with lower sICAM-1 and has been associated with lower risk of type I diabetes[30], while the minor allele of rs5498 (glutamic acid) is correlated with higher sICAM-1 levels and has been associated with lower risk of asthma [11] (MIM 600807), inflammatory bowel disease [31] (MIM 266600) and type I diabetes [32] (MIM 222100). Furthermore, it has been demonstrated *in vitro* that this SNP affects ICAM-1 mRNA splicing pattern and apoptosis in human peripheral blood mononuclear cells [33]. It is also noteworthy that sICAM-1 has been shown to inhibit insulitis and onset of autoimmune diabetes in a mouse model of type I diabetes [34] whereas ICAM1 itself was proven to be crucial to the priming of T cells against beta cells [35].

The most striking result of this report is the association between sICAM-1 levels and rs507666, a SNP intronic to the ABO gene. The ABO gene encodes glycosyltransferase enzymes which transfer specific sugar residues to a precursor substance, the H antigen. There are three major alleles at the ABO locus: A, B and O. Variation at the ABO locus is remarkable in that these alleles encode enzymes with different specificities as well as activities. The A allele encodes the enzyme alpha1→3 N-acetylgalactosamyl-transferase which forms the A antigen from the H antigen. The A allele (as well as the B and O alleles) is itself heterogeneous and comprises several subgroups, of which A1 and A2 are the most important. As compared to A1, the A2 allele has 30–50 fold less A transferase activity [36]. The B allele encodes the enzyme alpha1→3 galactosyltransferase which forms the B antigen from the H antigen. The O allele does not produce an active enzyme [37]. Consistent with the A antigen being associated with lower sICAM-1 concentrations and with the A1 allele having 30–50 fold more A transferase activity than the A2 allele, the A1 allele is associated with the lowest sICAM-1 concentrations while the A2 allele is associated with low concentrations as well, but still higher than the A1 allele (Table 5). Although we excluded the possibility of an analytical interference to explain the association, the exact mechanism linking histo-blood group antigen to sICAM-1 concentrations remains elusive. Among the different hypotheses, it remains possible that sICAM-1 bears the A antigen, a modification that might increase its clearance by increasing its affinity for its receptor(s) and/or decrease its secretion, perhaps by decreasing its affinity for the protease(s) producing sICAM-1 from membrane-bound ICAM1. Alternatively, lower sICAM-1 concentrations might be the result of the presence of the A antigen on its receptor(s) and/or protease(s).

ABO histo-blood group phenotype has been linked to a plethora of diseases, including infectious diseases, cancers and vascular diseases [38]. Particularly interesting is the association of non-O histo-blood groups — and group A in particular [39],[40] — with a higher risk of myocardial infarction, peripheral vascular disease, strokes and venous thromboembolism [41] (MIM 188050). While this phenomenon is partially explained by higher concentrations of the coagulation factors vonWillebrand and VIII (presumably because of decreased clearance) [42], the exact mechanism is not entirely understood. Underlining the complex nature of the biological processes involved, the A1 group (rs507666) is associated with lower levels of sICAM-1, a (positive) predictor of vascular diseases in epidemiological studies [5], [6], [43]–[46]. Among potential explanations as to this apparent disparity, it is possible that decreased sICAM-1 leads to increased adhesion of leukocytes on endothelial surface and therefore increased vascular inflammation, an important component of atherosclerosis [47]. Moreover, because group A individuals have been shown to have higher blood cholesterol [48] and coagulability [42], the decrease in sICAM-1 seen in these individuals could be offset by the increased susceptibility to vascular diseases conferred by these risk factors, even if sICAM-1 mechanistically causes these diseases. Alternatively, sICAM-1 might merely be a marker of increased inflammation and coagulation [49], both risk factors for vascular diseases. Also of special interest, group A antigen carriers have been recognized as having a higher risk of suffering from severe malaria when infected by *Plasmodium falciparum* [50]. *Plamodium* infected erythrocytes express a receptor (PfEMP-1) that binds specifically to cell-surface group A and B antigen as well as ICAM-1 [51], a major step in the sequestration of infected erythrocytes leading to the clinical complications of severe and cerebral malaria. The lower concentrations of sICAM-1 found in A1 group carriers could therefore be hypothesized to contribute to this higher risk either directly, if sICAM-1 can inhibit the sequestration process, or indirectly, if sICAM-1 levels reflect differences in the processing of the ICAM1 receptor itself.

Several limitations warrant discussion. First, this study was conducted in Caucasian women. It is therefore difficult to generalize our results to other ethnicities or to men. Second, effect estimates derived from this study might be higher than in other populations as these are initial findings and because of the winner's curse [52]. Third, although we were able to rule out a technical artifact as the cause of our results, no mechanistic link is identified to explain the association between ABO histo-blood groups and sICAM-1. In particular, one pending question is whether or not ICAM-1 bears any ABO antigen at all.

In this report, we demonstrate that sICAM-1 concentrations are associated with genetic variation at the ABO and ICAM1 loci in women. To our knowledge, this represents the first published genetic evidence that ABO may have a regulatory role on an inflammatory mediator, a finding with potential implication on a diverse array of immune-mediated disorders. Especially interesting is the fact that both ABO and ICAM1 have been previously related to vascular disease and malaria, two major causes of mortality and morbidity worldwide. The current study indicates a genetic link between histo-blood group antigen and inflammatory adhesion processes, providing the basis for physiological studies of this interaction.

## Supporting Information

Table S1 Clinical Characteristics of the Samples Used.
(0.04 MB DOC)

Table S2 Major and Minor Alleles.
(0.04 MB DOC)
