# Supplementary material for: Novel Association of ABO Histo-Blood Group Antigen with Soluble ICAM-1: Results of a Genome-Wide Association Study of 6,578 Women
Source: PLoS Genet. 2008 Jul 4;4(7):e1000118. doi: 10.1371/journal.pgen.1000118 (PMC2432033; doi:10.1371/journal.pgen.1000118)
Supplement: Table S1 — Clinical Characteristics of the Samples Used. (0.04 MB DOC) [file pgen.1000118.s001.doc]

| Table S1: Clinical characteristics of the samples used. | | | |  |
| --- | --- | --- | --- | --- |
|  |  |  |  |  |
|  | WGHS-1  (n=4570) | WGHS-2  (n=2008) | Combined  (n=6578) | P-value^a^ |
| Age (yrs.) | 54.1 (6.7) | 54.5 (6.9) | 54.2 (6.8) | 0.05 |
| BMI (kg/m^2) | 25.6 (4.9) | 25.8 (4.8) | 25.6 (4.8) | 0.20 |
| Menopause | 51.6% | 54.0% | 52.4% | 0.08 |
| HRT^b^ | 44.3% | 44.2% | 44.2% | 1.00 |
| Smoking | 11.9% | 11.6% | 11.8% | 0.76 |
| sICAM-1 (umol/L) | 348.7 (81.1) | 351.7 (78.2) | 349.6 (80.2) | 0.15 |
| Results are given as mean (standard deviation), as appropriate.  ^a^P-values were obtained using 2-tailed Student t-tests for continuous variables and Chi-square tests for dichotomous traits, comparing WGHS-1 to WGHS-2.  ^b^HRT: Hormone replacement therapy use. | | | | |
