# Supplementary material for: Novel Association of ABO Histo-Blood Group Antigen with Soluble ICAM-1: Results of a Genome-Wide Association Study of 6,578 Women
Source: PLoS Genet. 2008 Jul 4;4(7):e1000118. doi: 10.1371/journal.pgen.1000118 (PMC2432033; doi:10.1371/journal.pgen.1000118)
Supplement: Table S2 — Major and Minor Alleles. (0.04 MB DOC) [file pgen.1000118.s002.doc]

| Table S2: Major and Minor Alleles | | |
| --- | --- | --- |
|  |  |  |
|  | Major Allele | Minor Allele |
| rs10409243 | A | G |
| rs1799969 | G | A |
| rs2075741 | C | G |
| rs2116941 | C | A |
| rs2278442 | A | G |
| rs2304237 | A | G |
| rs281437 | C | T |
| rs281440 | A | G |
| rs3093030 | G | A |
| rs500498 | G | A |
| rs505922 | A | G |
| rs507666 | G | A |
| rs5498 | A | G |
| rs657152 | C | A |
| rs687289 | G | A |
| rs687621 | A | G |
| rs7256672 | A | C |
| rs7258015 | A | G |
| rs8111930 | G | A |
| rs923366 | G | A |
